# Correction: Fully-automated and field-deployable blood leukocyte separation platform using multi-dimensional double spiral (MDDS) inertial microfluidics

**DOI:** 10.1039/d3lc90038j

**Published:** 2023-04-06

**Authors:** Hyungkook Jeon, Bakr Jundi, Kyungyong Choi, Hyunryul Ryu, Bruce D. Levy, Geunbae Lim, Jongyoon Han

**Affiliations:** a Research Laboratory of Electronics, Massachusetts Institute of Technology (MIT) Cambridge MA 02139 USA jyhan@mit.edu; b Department of Mechanical Engineering, Pohang University of Science and Technology (POSTECH) 77 Cheongam-Ro, Nam-Gu Pohang Gyeongbuk 37673 Republic of Korea; c Division of Pulmonary and Critical Care Medicine, Brigham and Women's Hospital and Harvard Medical School Boston MA 02115 USA; d Department of Electrical Engineering and Computer Science, Massachusetts Institute of Technology (MIT) Cambridge MA 02139 USA; e Department of Biological Engineering, Massachusetts Institute of Technology (MIT) Cambridge MA 02139 USA

## Abstract

Correction for ‘Fully-automated and field-deployable blood leukocyte separation platform using multi-dimensional double spiral (MDDS) inertial microfluidics’ by Hyungkook Jeon *et al.*, *Lab Chip*, 2020, **20**, 3612–3624, https://doi.org/10.1039/D0LC00675K.

On page 3614, in the section “3.1. Design of the multi-dimensional double spiral (MDDS) device” there is an error in the sentence “Generally (for moderate flow rate conditions with a constraint of the Dean number, De = *R*_c_(*D*_h_/2*r*)^1/2^ < 75, where *δ* = *D*_h_/2*r* and *r* represent the curvature ratio and the average radius of curvature of the channel, respectively),^42^ in the case of small CRs (<0.07), the net lift force applied to particles is negligible compared to the Dean drag force, resulting in the circulating motion of particles without focusing (the non-focusing mode).^40,41^” This sentence should read “Generally (for moderate flow rate conditions with a constraint of the Dean number, De = *R*_c_(*D*_h_/2*r*)^1/2^ < 75, where *δ* = *D*_h_/2*r* and *r* represent the curvature ratio and the average radius of curvature of the channel, respectively),^42^ in the case of small CRs (<0.01), the net lift force applied to particles is negligible compared to the Dean drag force, resulting in the circulating motion of particles without focusing (the non-focusing mode).^40,41^”

This change does not affect any of the results or conclusions of the article.

The Royal Society of Chemistry apologises for these errors and any consequent inconvenience to authors and readers.

## Supplementary Material

